# Ketone ester supplementation blunts overreaching symptoms during endurance training overload

**DOI:** 10.1113/JP277831

**Published:** 2019-05-22

**Authors:** Chiel Poffé, Monique Ramaekers, Ruud Van Thienen, Peter Hespel

**Affiliations:** ^1^ Exercise Physiology Research Group Department of Movement Sciences KU Leuven Leuven Belgium; ^2^ Bakala Academy‐Athletic Performance Center KU Leuven Leuven Belgium

**Keywords:** ketone, overreaching and overtraining, exercise recovery, GDF15

## Abstract

**Key points:**

- Overload training is required for sustained performance gain in athletes (functional overreaching). However, excess overload may result in a catabolic state which causes performance decrements for weeks (non‐functional overreaching) up to months (overtraining).
- Blood ketone bodies can attenuate training‐ or fasting‐induced catabolic events. Therefore, we investigated whether increasing blood ketone levels by oral ketone ester (KE) intake can protect against endurance training‐induced overreaching.
- We show for the first time that KE intake following exercise markedly blunts the development of physiological symptoms indicating overreaching, and at the same time significantly enhances endurance exercise performance.
- We provide preliminary data to indicate that growth differentiation factor 15 (GDF15) may be a relevant hormonal marker to diagnose the development of overtraining.
- Collectively, our data indicate that ketone ester intake is a potent nutritional strategy to prevent the development of non‐functional overreaching and to stimulate endurance exercise performance.

**Abstract:**

It is well known that elevated blood ketones attenuate net muscle protein breakdown, as well as negate catabolic events, during energy deficit. Therefore, we hypothesized that oral ketones can blunt endurance training‐induced overreaching. Fit male subjects participated in two daily training sessions (3 weeks, 6 days/week) while receiving either a ketone ester (KE, *n* = 9) or a control drink (CON, *n* = 9) following each session. Sustainable training load in week 3 as well as power output in the final 30 min of a 2‐h standardized endurance session were 15% higher in KE than in CON (both *P* < 0.05). KE inhibited the training‐induced increase in nocturnal adrenaline (*P* < 0.01) and noradrenaline (*P* < 0.01) excretion, as well as blunted the decrease in resting (CON: −6 ± 2 bpm; KE: +2 ± 3 bpm, *P* < 0.05), submaximal (CON: −15 ± 3 bpm; KE: −7 ± 2 bpm, *P* < 0.05) and maximal (CON: −17 ± 2 bpm; KE: −10 ± 2 bpm, *P* < 0.01) heart rate. Energy balance during the training period spontaneously turned negative in CON (−2135 kJ/day), but not in KE (+198 kJ/day). The training consistently increased growth differentiation factor 15 (GDF15), but ∼2‐fold more in CON than in KE (*P* < 0.05). In addition, delta GDF15 correlated with the training‐induced drop in maximal heart rate (*r* = 0.60, *P* < 0.001) and decrease in osteocalcin (*r* = 0.61, *P* < 0.01). Other measurements such as blood ACTH, cortisol, IL‐6, leptin, ghrelin and lymphocyte count, and muscle glycogen content did not differentiate KE from CON. In conclusion, KE during strenuous endurance training attenuates the development of overreaching. We also identify GDF15 as a possible marker of overtraining.

## Key points

- Overload training is required for sustained performance gain in athletes (functional overreaching). However, excess overload may result in a catabolic state which causes performance decrements for weeks (non‐functional overreaching) up to months (overtraining).
- Blood ketone bodies can attenuate training‐ or fasting‐induced catabolic events. Therefore, we investigated whether increasing blood ketone levels by oral ketone ester (KE) intake can protect against endurance training‐induced overreaching.
- We show for the first time that KE intake following exercise markedly blunts the development of physiological symptoms indicating overreaching, and at the same time significantly enhances endurance exercise performance.
- We provide preliminary data to indicate that growth differentiation factor 15 (GDF15) may be a relevant hormonal marker to diagnose the development of overtraining.
- Collectively, our data indicate that ketone ester intake is a potent nutritional strategy to prevent the development of non‐functional overreaching and to stimulate endurance exercise performance.

## Introduction

Endurance athletes intermittently participate in overload training (e.g. training camps) or competition (e.g. multi‐stage cycling races) with the express purpose of eliciting physiological responses that are crucial for sustained performance gain. Training overload, however, must be consistently well balanced with recovery, allowing physiological repair mechanisms to produce beneficial adaptations that eventually yield performance gains (e.g. functional overreaching). Inadequate recovery, with or without other stress factors, such as sleep deprivation, negative energy balance, disease or mental fatigue, eventually can result in a maladaptive catabolic state requiring days to weeks (non‐functional overreaching), or even months (overtraining) to fully recover (Meeusen *et al*. 2013). Because sufficient recovery will only result in performance improvements during functional overreaching, prevention of non‐functional overreaching is pivotal in training management.

The primary indication of overreaching is a stagnation or decrease in training‐specific performance (Hooper *et al*. 1995; Urhausen & Kindermann, 2002). However, this is often preceded by numerous other symptoms, such as mood disturbances (Morgan *et al*. 1987; Killer *et al*. 2017) and dysregulation in various physiological systems, including the autonomic nervous system (Lehmann *et al*. 1998), immunity (Fry *et al*. 1994b) and energy metabolism (Lombardi *et al*. 2012). Nevertheless, previous studies have failed to identify consistent physiological outcomes (Urhausen & Kindermann, 2002; Cadegiani & Kater, 2017) that can be used to predict overtraining. In fact, the pathophysiology of overtraining remains poorly understood, which impairs the design of optimal preventive interventions (Armstrong & Vanheest, 2002; Kreher & Schwartz, 2012).

Numerous studies have focused on the use of post‐exercise nutrition to facilitate recovery between training sessions and thereby counteract overtraining (Kreider *et al*. 2010; Hawley *et al*. 2011). Amongst various nutritional interventions, protein–carbohydrate co‐ingestion is recognized to be the best strategy to enhance recovery by stimulating both muscle glycogen resynthesis (van Loon *et al*. 2000) and muscle repair (Breen *et al*. 2011). However, protein plus carbohydrate ingestion is insufficient to prevent overtraining (Achten *et al*. 2004; Halson *et al*. 2004; Witard *et al*. 2011; D'Lugos *et al*. 2016; Svendsen *et al*. 2016). Other nutritional interventions, such as antioxidant intake to protect against exercise‐induced oxidative stress, fail to negate overtraining (Gleeson & Bishop, 2000; Meeusen & Watson, 2007). In contrast, consistent antioxidant intake may even inhibit beneficial training adaptations (Merry & Ristow, 2016).

Some recent publications have stimulated interest in the use of exogenous ketone supplements as a novel fuelling strategy to modulate metabolic responses both during (Cox *et al*. 2016; Leckey *et al*. 2017) and after exercise (Holdsworth *et al*. 2017; Vandoorne *et al*. 2017). Ketone bodies, namely d‐β‐hydroxybutyrate (d‐βHB), acetoacetate (AcAc) and acetone, are fatty acid‐derived compounds that can serve as an alternative energy substrate for active metabolic tissues including brain (Owen *et al*. 1967), heart (Aubert *et al*. 2016) or skeletal muscles (Balasse & Féry, 1989) under conditions of metabolic stress (Johnson *et al*. 1969; Cahill, 1976). Aside from their role in energy supply, ketone bodies also can play a role in metabolic regulation by inhibiting muscle proteolysis (Thomsen *et al*. 2018) and glucose depletion (Robinson & Williamson, 1980), and stimulation of muscle regeneration or remodelling by enhancing satellite cell activation and differentiation (Zou *et al*. 2016). Moreover, ketone bodies directly regulate a range of putative factors involved in the development of overtraining, such as autonomic neural output, inflammation and oxidative stress (Kimura *et al*. 2011; Puchalska & Crawford, 2017). These actions clearly indicate a potential role for ketone bodies in prevention of overtraining, but the dietary conditions required to elevate blood ketone levels, by a sustained low‐carbohydrate high‐fat diet, are detrimental to endurance exercise performance (Cox & Clarke, 2014).

However, exogenous ketone supplements recently emerged as a novel approach to induce ketosis. Exogenous supplements are available either in the form of ketone salts or ketone esters (Cox *et al*. 2016; Brownlow *et al*. 2017; Leckey *et al*. 2017), but ketone esters allow blood ketones to reach ∼3‐fold higher levels than salts, with less incidence of gastrointestinal problems (Stubbs *et al*. 2017; Sansone *et al*. 2018). Recent studies in our and other laboratories have shown that post‐exercise ingestion of the ketone monoester (*R*)‐3‐hydroxybutyl (*R*)‐3‐hydroxybutyrate (KE) stimulates markers of protein synthesis and potentially also muscle glycogen repletion following exercise (Holdsworth *et al*. 2017; Vandoorne *et al*. 2017). However, these experiments have only looked at the acute effects following a single, high‐intensity exercise bout. The effects of long‐term ketone ester intake in training and recovery remain unknown. Given the evidence that d‐βHB infusion antagonizes starvation‐induced catabolic processes (Sherwin *et al*. 1975; Pawan & Semple, 1983), it is reasonable to postulate that ketosis induced by consistent exogenous ketone intake during strenuous training may alleviate detrimental catabolic events.

Therefore, we performed a double‐blind, placebo‐controlled study to assess the effect of KE during strenuous endurance training with the express purpose of producing a state of non‐functional overreaching. We hypothesized that KE can attenuate training‐induced pathophysiological effects leading up to non‐functional overreaching, and eventually enhance endurance exercise performance.

## Methods

### Ethical approval and subjects

Twenty healthy, physically active males were recruited to participate in this study, which was approved by the KU Leuven Biomedical Ethics Committee (B322201733747), and conforms to the *Declaration of Helsinki*. Potential subjects were screened using a medical questionnaire and a physical examination, including a resting ECG, prior to involvement in the study. From the initial 20 recruits, one subject did not complete the study due to adverse reactions to the protein–carbohydrate drinks prescribed by the study protocol, and another withdrew for reasons unrelated to the study protocol. Eighteen subjects eventually completed the full study protocol and were included in the final data analyses (for subject characteristics see Table 1). All subjects were regularly involved in sports and physical activity at a rate of 4.8 ± 0.4 h/week (mean ± SEM), but none were consistently engaged in cycling. Throughout the entire study period, subjects were instructed to refrain from strenuous exercise other than prescribed by the study protocol. All subjects were informed of the content and potential risks involved with the experimental procedures before providing their written consent.

**Table 1:** Subject characteristics

	Control	Ketone ester
Age (years)	21.2 ± 2.9	21.4 ± 2.4
Height (m)	1.81 ± 0.04	1.80 ± 0.04
Body mass (kg)	74.6 ± 10.5	72.8 ± 6.5
V˙O2 max (ml/kg/min)	55.3 ± 6.1	55.9 ± 5.5

Values are mean ± SD and represent baseline characteristics of the subjects receiving either control (*n* = 9) or ketone ester (*n* = 9).

### Preliminary testing and subject randomization

Two weeks before baseline measurements, the subjects completed three preliminary sessions with a 48‐h rest interval in between. During the first visit, subjects performed a maximal incremental exercise test on a bicycle ergometer (Avantronic Cyclus II, Leipzig, Germany) to determine their V˙O2 max . Initial workload was set at 70 W, followed by 25‐W increments per minute, until volitional exhaustion. Respiratory gas exchange was measured continuously during the test (Cortex Metalyzer II, Leipzig, Germany) and the highest oxygen uptake measured over a 30 s period was defined as the maximal oxygen uptake rate (V˙O2 max ). During the second and third session, subjects were familiarized with the exercise testing procedures to undergo in the experimental sessions. Subjects started with 10 min of warming up (5 min at 100 W, 5 min at 150 W) followed by a 30 min simulated time‐trial (TT_30min_) on a cycling ergometer (Avantronic Cyclus II). They were instructed to maintain their cadence between 80 and 100 rpm, and to adjust the resistance at 5 min intervals from t5 to t25, and every min from t25 to t30 to develop the highest possible mean power output (W) over 30 min. Following 15 min of active recovery by cycling at 50 W, the subjects performed a 90 s all‐out sprint (90S) on a self‐constructed isokinetic cycling ergometer (Koninckx *et al*. 2008, 2010) with cadence fixed at 90 rpm. Following familiarization, the subjects were pair‐matched to obtain two groups with similar distributions for V˙O2 max , mean power outputs in TT_30min_ and 90S, training history (hours/week), and body mass and height. The matched pairs were then randomly split into two experimental groups by an investigator who was otherwise not involved in the trial.

### General experimental design

After randomization the subjects were enrolled in a fully controlled 3‐week intensive cycling training programme which was designed with the express purpose of inducing a state of non‐functional overreaching. During the training period the subjects received either a ketone ester drink (KE, *n* = 9) or a corresponding control drink (CON, *n* = 9). Following the training period, the subjects were followed‐up during a tapering week without training.

### Training intervention

**Figure 1:**
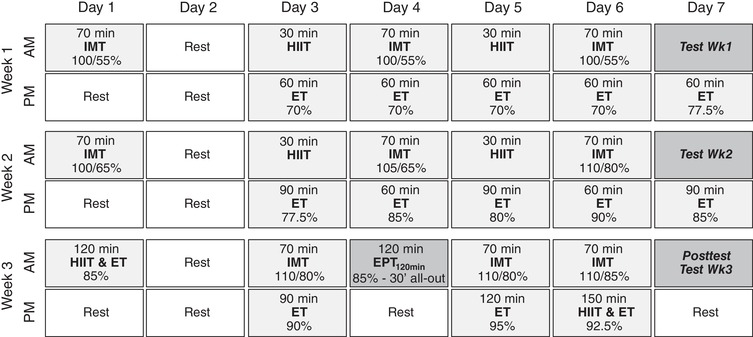
Overview of the training programme HIIT, high‐intensity interval training: 30 s all‐out sprints interspersed by 4.5 min active recovery intervals (50 W). The number of sprints was increased from 4 in week 1, to 5 in week 2, and 6 in week 3. IMT, intermittent endurance sessions. Week 1 and 2: 5 × 6 min with 8 min recovery. Week 3: 5 × 8 min with 6 min recovery, ET, constant‐load endurance training sessions. EPT_120min_, 120 min endurance performance test. Training intensities for IMT and ET are expressed as a percentage of the mean power output effected during the 30 min time‐trial (TT_30min_) in the pre‐test.

A detailed overview of the training programme is shown in Fig. 1. The 3‐week overload‐training programme (28 training sessions) consisted of a combination of high‐intensity interval training (HIIT), intermittent endurance training (IMT) and constant‐load endurance training (ET) sessions. All training sessions were performed in the laboratory on an electromagnetically braked ergometer and under the careful supervision of the investigators. The HIIT and IMT sessions were done on calibrated cycling ergometers (Avantronic Cyclus II) in order to be able to monitor the power output–heart rate relationship (W). The ET sessions were alternately performed on the same calibrated ergometers or on routine cycling ergotrainers (Tacx Neo Smart, Wassenaar, The Netherlands). The workload in ET sessions on the routine ergotrainers was based on the heart rate–power relationship obtained from the sessions on the calibrated ergometers (2–3×/week). During the ET sessions, subjects cycled at a continuous load (70–95% of mean power output TT_30min_) for 60–150 min. HIIT sessions consisted of 30 s maximal sprints at a cadence fixed at 100 rpm, interspersed by 4 min 30 s active recovery intervals at 50 W. The number of sprints was increased from four in week 1, five in week 2, to six in week 3. Subjects were given verbal encouragement to perform maximally during each sprint. IMT sessions consisted of five 6‐min bouts at 100–110% of mean power output taken from TT_30min_, separated by 8 min of lower intensity at 55–85%. During the final week, the duration of the high‐intensity bouts was increased to 8 min, whilst decreased to 6 min for the lower intensity bouts. Exercise intensities of IMT and ET were calculated relative to the mean power output effected during TT_30min_ in the pre‐test. The intensity of each training session is given in Fig. 1. If a subject failed to maintain a cadence ≥ 70 rpm at the prescribed workload, the workload was decreased to the level that allowed the subjects to return and maintain a cadence ≥70 rpm.

### Post‐exercise nutritional intervention

Subjects from both experimental groups received a 500 ml high‐dose protein–carbohydrate drink (Table 2) 30 min after each exercise session. In addition, immediately following each session and 30 min before sleep, KE subjects received 25 g of ketone ester [96% (*R*)‐3‐hydroxybutyl (*R*)‐3‐hydroxybutyrate] to elevate post‐exercise circulating plasma ketone concentrations, as previously shown by our lab (Vandoorne *et al*. 2017). The ketone ester supplements were purchased from TdeltaS Ltd (Thame, UK). Subjects in CON received an isocaloric drink (CON) containing 16.4 g pure medium‐chain triglycerides (Now Foods, Bloomingdale, IL, USA). To equalize the taste and appearance of CON and KE drinks, 1 mm bitter sucrose octaacetate (Sigma‐Aldrich, Bornem, Belgium) was added to the CON, whilst a red colorant (AVEVE Bloem, Merksem, Belgium) was added to both the drinks to obtain similar appearance. Subjects received 50 ml of diet coke for mouth rinsing immediately following the KE or CON drink to improve palatability (Leckey *et al*. 2017). Capillary blood samples from the earlobe were obtained before and after exercise and 30 min following ingestion of the supplements to assess circulating blood β‐hydroxybutyrate concentrations (Glucomen Lx plus‐meter with Lx β‐ketone strips, Menarini Diagnostics, Firenze, Italy) during IMT sessions on days 6, 13 and 20 of the training period.

**Table 2:** Composition of protein–carbohydrate recovery drinks

	Protein‐carbohydrate recovery mixture
Total carbohydrates	60.6 g
Maltodextrin	30.6 g
Sucrose	20.0 g
Fructose	10.0 g
Total protein	31.0 g
Non‐essential amino acids	15.9 g
Essential amino acids	15.1 g
Of which leucine	3.13 g

Composition of protein–carbohydrate drinks given 30 min following each training session. Data represent grams of ingredients per 500 ml drink.

### Experimental trials

Before (pre‐test), during (days 7 and 14) and after (post‐test) the training period, as well as during the recovery phase (3 and 7 days later), the subjects participated in an experimental session involving a TT_30min_ and 90 s isokinetic sprint. On the evening before each session, they received a standardized carbohydrate‐rich dinner (∼5400 kJ; 69% carbohydrate, 16% fat, 15% protein). Next morning and after an overnight fast, a blood sample was collected from both an earlobe (capillary blood) and from an antecubital vein (Venoject, Tokyo, Japan). The subjects then received a standardized breakfast (∼2700 kJ; 71% carbohydrate, 15% fat, 14% protein). Following a 1.5 h rest in a comfortable chair they warmed up for 10 min at incremental workloads corresponding to 70% (5 min) and 85% (5 min) of their average power output recorded during TT_30min_ in the last familiarization session. Thereafter, they performed a TT_30min_ in which they aimed for the highest possible mean power output. During the first 5 min (t0–t5), the workload was set equal to the average power obtained during the last familiarization session. From t5 to t25, subjects were allowed to adjust the workload at 5‐min intervals according to their subjective perception of fatigue. From t25 to t30, 1‐min adjustments were allowed to facilitate full exhaustion by the end of TT_30min_. Subjects were allowed to drink water *ad libitum* and received online feedback about the time to completion. On completion of the TT_30min_, they recovered for 15 min by cycling at 50 W, followed by the 90S. On day 18 of the training period, a 120 min endurance exercise performance test (EPT_120min_) was included to assess endurance performance (Jeukendrup *et al*. 1996). This session consisted of a 90 min preload (cycling at 85% of the mean power output effected during TT_30min_ in the pre‐test) to induce fatigue, followed by an all‐out 30 min time trial. During these tests, heart rate was monitored continuously (Polar RS800CX, Kempele, Finland), while blood lactate concentration was measured (Lactate Pro2, Arkray, Japan) in a capillary blood sample from the earlobe at 5‐min intervals during TT_30min_, and 3 min after completion of 90S. Ratings of perceived exertion (RPE, 6–20 Borg Scale; Borg, 1990) were recorded immediately after completion of TT_30min_, 90S and EPT_120min_. Power outputs were blinded to the subjects during all tests. Standardized verbal encouragement was provided only during 90S. During the pre‐ and post‐test, an additional blood sample was obtained from an antecubital vein (Venoject) immediately before and after the TT_30min_.

### Body composition and anthropometry

Whole‐body dual‐energy X‐ray absorptiometry (DXA) scans (Discovery W, Hologic Inc., Bedford, MA, USA) were made during both the pre‐test and the post‐test. Scans were performed in the fasted state and at the same time of the day during both sessions to minimize measurement errors (Bone & Burke, 2016). Output parameters considered were whole‐body bone mineral content (BMC) and density (BMD), and percentage body fat and lean soft tissue mass. All scans were performed by a single certified technician, and subject positions over the different sessions were standardized according to the manufacturer's recommendations. The densitometer was calibrated daily against a spinal phantom to account for potential day‐to‐day variability. Following the DXA scan, skinfold thickness was measured at 12 sites (biceps, triceps, subscapular, supra‐iliac, midaxillary, iliac‐crest, abdomen, chin, anterior thigh, posterior thigh, lateral calf and medial calf) according to standard procedures.

### Subjective feelings of appetite, gastrointestinal discomfort and recovery–stress state

During each experimental session, subjects completed three questionnaires to assess their: (i) appetite sensations; (ii) gastrointestinal discomfort; and (iii) recovery–stress state. Appetite was assessed immediately before breakfast using a validated 0–10 Likert visual analog scale (VAS), adapted from Woods *et al*. (2018). Subjects were provided with four appetite or satiety questions (‘How hungry do you feel?’, ‘How full do you feel?’, ‘How satisfied do you feel?’, ‘How much do you think you could eat now?’). Gastrointestinal discomfort was rated after completion of breakfast by means of a 0–8 Likert scale questionnaire adapted from Pfeiffer *et al*. (2009). The questionnaire comprised three sections, i.e. upper abdominal problems (reflux, bloating, nausea, vomiting); lower abdominal problems (cramps, flatulence, abdominal pain, diarrhoea); and systemic problems (dizziness, headache, muscle cramp, urge to urinate). Subsequently, the Recovery‐Stress Questionnaire for Athletes (RESTQ‐76 Sport questionnaire) was administered to assess subjects’ recovery–stress state and to classify whether they became overreached or overtrained (Kellmann & Kallus, 2001). In accordance with a previous study, the recovery–stress balance was calculated by subtracting the total recovery score (∑9 recovery subscales) from the total stress score (∑10 stress subscales) (Coutts *et al*. 2007). High scores in the recovery‐associated scales represent adequate recovery, while high scores for the stress‐oriented subscales represent intense subjective strain.

### Nutritional control

Nutritional intake was monitored via an online dietary platform (Mijn Eetmeter, Stichting Voedingscentrum Nederland; https://mijn.voedingscentrum.nl). The user modalities and dietary recording procedure were explained in detail to the subjects following the last familiarization session. Diaries were obtained during two subsequent days at the beginning (days 3–4), mid (days 12–13) and at the end (days 19–20) of the training period. Diaries were analysed for both macronutrient and energy intake.

### Muscle biopsy procedure and muscle glycogen content

During the pre‐ and post‐test, a percutaneous needle biopsy (100–200 mg) was obtained under local anaesthesia (2% xylocaine without adrenaline, 1 ml subcutaneously) before and immediately after the TT_30min_ from the m. vastus lateralis using a 5‐mm Bergström‐type needle. Biopsies during the pre‐ and post‐test were taken from the left and right leg, respectively, while pre‐ and post‐exercise biopsies were obtained through the same incision, but with the needle pointing distal *vs*. proximal, respectively (Van Thienen *et al*. 2014). Part of the muscle sample was immediately frozen in liquid nitrogen and stored at −80°C for assay of muscle glycogen content at a later date. Muscle glycogen content was determined as glucose residues after acid hydrolysis using a standard enzymatic fluorometric assay (Lowry & Passoneau, 1972).

### Urine sampling and analysis

Nocturnal urine was collected over 12 h (20.00–22.00 h until 08.00–10.00 h) the night before each experimental session in flasks prepared with 10 ml hydrochloric acid. Total urinary output volume was noted, and urinary ketone concentration was measured using ketone reagent strips (Ketostix, Ascensia Diabetes Care). An aliquot of the urine sample was stored at −80°C until assayed in a single run for adrenaline and noradrenaline concentration using a commercially available enzyme linked immunosorbent assay (ELISA) (BA E‐5400, LDN, Nordhorn, Germany).

### Analysis of blood samples

Capillary blood samples from the earlobe were immediately analysed for blood d‐βHB (Glucomen Lx plus‐meter with Lx β‐ketone strips, Menarini Diagnostics). d‐βHB measurements were performed by an investigator who was otherwise not involved in the experimental testing to ensure double‐blindness. Venous blood samples were collected into vacuum tubes containing either EDTA or lithium heparin or Silica Clot Activator (BD Vacutainer). Tubes were centrifuged (1500 rpm for 10 min at 4°C) and the supernatant was stored at −20°C until later analysis. Commercially available ELISAs were performed to determine leptin, total ghrelin, growth differentiation factor 15 (GDF15) and total osteocalcin in serum, while IL‐6 levels were determined in EDTA plasma (Leptin: BMS2039INST, Thermo Fisher Scientific, Waltham, MA, USA; Total Ghrelin: EZGRT‐89K, Merck, Darmstadt, Germany; GDF15: DGD150, R&D, Minneapolis, MN, USA; Total osteocalcin: KAQ1381, Thermo Fisher Scientific; IL‐6: HS‐600B, R&D). Cortisol and ACTH levels were assayed using electrochemiluminescence immunoassays (ECLIAs) in serum and EDTA plasma samples, respectively. TRAP5b activity was measured in serum by a direct capture enzyme‐immunoassay (no. 8036, TECOmedical, Sissach, Switzerland). Fasted whole blood samples obtained during the pre‐ and post‐test were analysed for the proportion of lymphocyte subset types, i.e. T‐cell (CD3+), T‐helper/inducer cell (CD4+), T‐suppressor/cytotoxic cell (CD8+) and CD4+/CD8+ cell count by flow cytometry. Plasma glucose levels were determined using a standard enzymatic fluorometric assay (Lowry & Passoneau, 1972). All assays were run in a single batch, which included all samples, according to the respective protocols supplied by the manufacturer.

### Statistical analysis

Statistical analyses were performed in R version 3.3.3 using the nlme package for mixed‐effects models and the stats package for unpaired *t* tests (R Development Core Team, Vienna, Austria). Differences between mean values over time and between conditions were analysed using a two‐way repeated‐measures analysis of variance (group × time). d‐βhb levels during the training sessions, as well as muscle glycogen content and blood glucose levels before and after TT_30min_ were assessed by a three‐way repeated‐measures analysis of variance (group × time × number of training/experimental session). Pearson correlation coefficients were calculated using percentage change scores unless otherwise stated. Holm–Sidak's multiple comparison test was used for *post hoc* analysis, when appropriate. Where relevant, Cohen's *d* was calculated as index of effect size (ES). Outliers were identified using the ROUT method in GraphPad Prism version 8.00 (GraphPad Software, La Jolla, CA, USA). If outliers were detected, statistics were performed both with and without the outlier and both results are included in the text. Statistical significance was defined as *P* < 0.05. Data are presented as mean ± SEM.

## Results

### Blood d‐βHB and urinary ketone excretion

**Figure 2:**
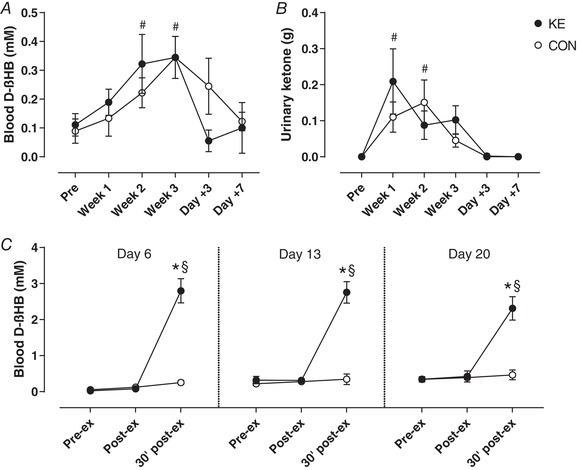
Effect of ketone ester supplementation on blood d‐βHB concentrations and urinary ketone excretion Data are mean ± SEM for fasted morning blood d‐βHB concentration (*A*) and nocturnal urinary ketone excretion (*B*) before (Pre) and at the end of weeks 1 (Week 1), 2 (Week 2) and 3 (Week 3) of the training period, and after 3 (Day +3) and 7 (Day +7) recovery days after training. During the training period the subjects received either control (○, *n* = 9) or ketone ester supplements (●, *n* = 9) immediately after each training session. *C*, blood d‐βHB concentrations before (Pre‐ex), and immediately (Post‐ex) and 30 min after (30’ post‐ex) the IMT sessions on days 6, 13 and 20. ^*^ *P* < 0.05 KE *vs*. CON at time points indicated; ^#^ *P* < 0.05 *vs*. PRE for both KE and CON; ^§^ *P* < 0.05 *vs*. pre‐ex for the indicated group.

In the pre‐test, fasted blood d‐βHB levels were similar between the groups at ∼0.1 mm. During the training period, fasted d‐βHB levels gradually increased, reaching peak levels at ∼0.35 mm in the post‐test (*P* < 0.01; Fig. 2 *A*). During the recovery week, blood d‐βHB levels returned towards baseline within 3 days. There were no differences between the groups at any time. Ketone bodies were undetectable (<0.05 g/l) in urine during the pre‐test (Fig. 2 *B*), yet transiently increased during weeks 1 and 2 (*P* < 0.01 and *P* < 0.001, respectively), irrespective of the experimental condition. During the training sessions, blood d‐βHB levels were low (∼0.1–0.3 mm) in both the groups before and immediately after exercise (Fig. 2 *C*). However, ketone ester intake immediately after exercise increased blood d‐βHB levels to 2.6 ± 0.2 mm within 30 min (30’ post‐ex in Fig. 2 *C*), whilst values were unchanged in CON (*P* < 0.001 *vs*. KE).

### Training load

**Figure 3:**
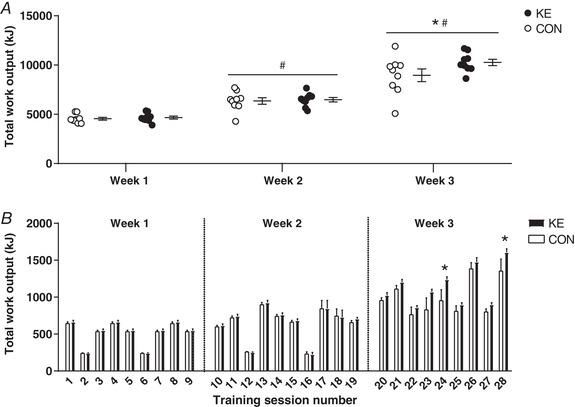
Effect of ketone ester supplementation on training workload *A*, individual data points together with means ± SEM representing total work output per week. *B*, means ± SEM for work output per training session in subjects receiving either control (○/bars, *n* = 9) or ketone ester (●/bars, *n* = 9) supplements. The subjects performed 28 training sessions over a 3‐week training period. ^*^ *P* < 0.05 KE *vs*. CON at time points indicated; ^#^ *P* < 0.05 *vs*. PRE for both KE and CON.

Total weekly training load progressively increased from ∼4600 kJ in week 1, to ∼6400 kJ in week 2 and ∼9600 kJ in week 3 (Fig. 3 *A*). Compared to their normal training volume (4.8 ± 0.4 h/week), this corresponds to a ∼120% increment in week 1, 165% in week 2 and a ∼3‐fold increment in week 3. Training load was similar between the groups in weeks 1 and 2, but was ∼15% higher in KE than in CON at week 3 [KE: 10266 ± 321 kJ *vs*. CON: 8962 ± 646 kJ, 95% confidence interval (CI): +237 to +2372 kJ, *P* < 0.05, *d* = 0.80]. Differences in work output between the groups were most explicit for the prolonged endurance training sessions (ET) at the end of the training period, with no significant differences for the HIIT and IMT sessions (Fig. 3 *B*).

### Exercise performance and peak lactate concentration

**Figure 4:**
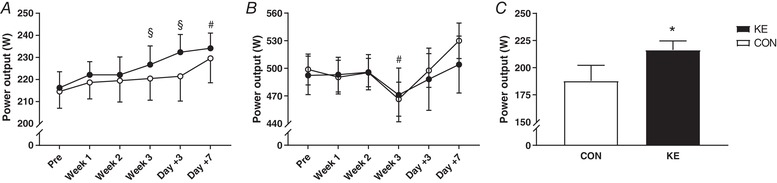
Effect of ketone ester supplementation on exercise performance Data are means ± SEM. *A* and *B*, mean power output during the 30 min simulated time‐trial (TT_30min_) (*A*) and in a 90 s all‐out cycling bout (90S) (*B*) before (Pre) and at the end of weeks 1 (Week 1), 2 (Week 2) and 3 (Week 3) of the training period, and after 3 (Day +3) and 7 (Day +7) days of recovery. *C*, mean power output in the final half an hour of a 120 min endurance performance test (EPT_120min_) on day 18 of the training period. Subjects received either control (○/open bars, *n* = 9) or ketone ester (●/filled bars, *n* = 9) during each training session. ^*^ *P* < 0.05 KE *vs*. CON; ^#^ *P* < 0.05 *vs*. PRE for both KE and CON; ^§^ *P* < 0.05 *vs*. PRE for indicated group.

Mean power outputs in TT_30min_ and 90S in the pre‐test were similar between the groups (TT_30min_: 216 ± 7 *vs*. 215 ± 8 W; 90S: 492 ± 20 *vs*. 499 ± 17 W for KE and CON, respectively). In KE, compared to the pre‐test mean power output in TT_30min_ was 4.9 ± 1.5% higher (95% CI: +3 to +18 W, *P* < 0.05, *d* = 0.47) in the post‐test, and 7.5 ± 1.7% (95% CI: +8 to +24 W, *P* < 0.001, *d* = 0.71) and 8.3 ± 2.1% (95% CI: +8 to +28 W, *P* < 0.001, *d* = 0.83) higher on day +3 and day +7, respectively (Fig. 4 *A*). Conversely, training did not improve TT_30min_ performance in CON (95% CI pre‐ *vs*. post‐test: −2 to +14 W, *P* > 0.05), except on day +7 (+7.0 ± 2.0%, 95% CI: +5 to +25 W, *P* < 0.001, *d* = 0.52). However, TT_30min_ mean power outputs were not significantly different between CON and KE at any time (95% CI for KE *vs*. CON during post‐test: −33 to +14 W, *P* > 0.05). Furthermore, mean power output during EPT_120min_ on day 18 was ∼15% higher in KE than in CON (KE: 216 ± 8 W *vs*. CON: 188 ± 14 W, 95% CI for KE *vs*. CON: +5 to +52 W, *P* < 0.05, *d* = 0.77; Fig. 4 *C*). Compared to the pre‐test, mean power output effected in 90S was stable in weeks 1 and 2, but decreased by 5.5 ± 1.4% in the post‐test (*P* < 0.05, *d* = 0.30; Fig. 4 *B*). Yet during the recovery period 90S power outputs returned to baseline by day +3. Performance in 90S was not significantly different between the groups at any time. Blood lactate levels following 90S peaked at ∼13–15 mm in the pre‐test as well as in weeks 1 and 2. However, in the post‐test peak blood lactate levels decreased by ∼5 mm (*P* < 0.001) to 9.8 ± 1.0 mm in CON and 8.2 ± 0.8 mm in KE. During the recovery period, blood lactate concentrations rapidly returned to baseline in CON (day +3: 13.2 ± 1.9 mm, *P* = 0.29 *vs*. PRE; day +7: 12.6 ± 1.5 mm, *P* = 0.10 *vs*. PRE), but not in the KE group (day +3: 10.9 ± 0.8 mm, *P* < 0.05 *vs*. PRE; day +7: 9.6 ± 1.1 mm, *P* < 0.01 *vs*. PRE).

### Heart rate and blood pressure

**Figure 5:**
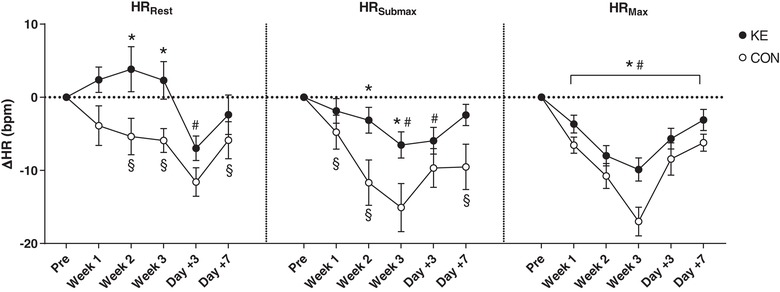
Effect of ketone ester supplementation on heart rate Data are means ± SEM and represent changes in resting (HR_Rest_), submaximal (HR_Submax_) and maximal (HR_Max_) heart rate before (Pre), and at the end of weeks 1 (Week 1), 2 (Week 2) and 3 (Week 3) of the training period, and after 3 (Day +3) and 7 (Day +7) recovery days. Subjects received either control (○, *n* = 9) or ketone ester supplements (●, *n* = 9) following each training session. ^*^ *P* < 0.05 KE *vs*. CON; ^#^ *P* < 0.05 *vs*. PRE for both KE and CON; ^§^ *P* < 0.05 *vs*. PRE for indicated group.

Resting heart rate (HR) was 62 ± 4 bpm in KE *vs*. 66 ± 2 bpm in CON (*P* = 0.73). In CON the training intervention gradually decreased resting HR to 60 ± 1 bpm in the post‐test (*P* < 0.05), and even further to 55 ± 2 bpm at day +3 (*P* < 0.001; Fig. 5 *A*). At day +7, resting HR was still ∼6 bpm lower than in the pre‐test (*P* < 0.05). In contrast, in KE resting HR was stable throughout the full training period, and consistently was ∼2–5 bpm higher than in CON (*P* < 0.05). Only on day +3, resting HR in KE was slightly lower than at baseline (*P* < 0.05). The training also substantially decreased HR during submaximal and maximal exercise. In CON submaximal and maximal HR on average decreased by ∼16 bpm (range: −5 to –28 bpm) from the pre‐test to the post‐test (*P* < 0.001; Fig. 5 *B*, *C*). Thus, submaximal HR during TT_30min_ was 167 ± 4 in the pre‐test, decreasing to 152 ± 4 bpm in the post‐test. By analogy, maximal HR taken from 90S dropped from 189 ± 2 to 172 ± 3 bpm (*P* < 0.001). In KE submaximal and maximal HRs also dropped during the training period, yet the drop was markedly smaller than in CON (*P* < 0.05 and *P* < 0.01 *vs*. CON, respectively). Submaximal HR dropped from 163 ± 3 in the pre‐test to 157 ± 3 bpm in the post‐test (*P* < 0.05). Corresponding maximal HRs were 189 ± 3 and 179 ± 2 bpm (*P* < 0.001). In both the groups the training‐induced suppression of submaximal and maximal HR was rapidly inverted during the recovery week. Within a week, submaximal HR returned to baseline in KE, but not in CON (*P* < 0.001). Clearly, ketone ester intake consistently blunted (over)training‐induced bradycardia both at rest and during submaximal and maximal exercise. Furthermore, the decrement of resting HR effected by the training intervention was associated with a drop of diastolic blood pressure from 68 ± 1 to 57 ± 3 mmHg in CON (*P* < 0.001), but not in KE (pre‐test 67 ± 2, post‐test 62 ± 2 mmHg, *P* = 0.20). Systolic blood pressures on average were ∼126 mmHg and were not altered by either training or ketone ester intake.

### Body composition and bone mineralization

Baseline values for body composition and bone mineralization were similar between the two experimental groups (Table 3). The training period decreased body fat percentage by 1.4 ± 0.2% (*P* < 0.001, *d* = 0.36) and sum of skinfolds by 7.3 ± 1.4 mm (*P* < 0.001, *d* = 0.28), while both lean mass and body weight remained unchanged irrespective of the experimental conditions. In KE, BMC from the pre‐test to the post‐test increased by 24 ± 10 g (*P* < 0.01, *d* = 0.07), whilst it was stable in CON (−6 ± 8 g, *P* = 0.53). BMD was unaffected by the experimental conditions.

**Table 3:** Effect of ketone ester supplementation on body composition and bone mineralization

	Control		Ketone ester	
	Pre	Post	Pre	Post
Body weight (kg)	72.9 ± 3.6	73.1 ± 3.2	72.1 ± 2.1	71.9 ± 2.2
Lean mass (kg)	60.4 ± 2.6	61.6 ± 2.4	61.1 ± 1.9	61.8 ± 1.9
Percent body fat (%)	13.1 ± 1.3	11.6 ± 1.1#	11.4 ± 0.9	10.1 ± 0.8#
Sum skinfolds (mm)	103.6 ± 10.6	95.3 ± 8.9#	87.1 ± 7.7	80.8 ± 7.2#
BMC (g)	2749 ± 133	2744 ± 130	2758 ± 106	2782 ± 112# ^,^ §
BMD (g/cm^2^)	1.19 ± 0.04	1.19 ± 0.04	1.23 ± 0.03	1.24 ± 0.03

Data are mean ± SEM for body weight, body composition and bone mineralization parameters measured by DXA scan, in subjects receiving either ketone ester supplements (*n* = 9) or control (*n* = 9). BMC, bone mineral content; BMD, bone mineral density. ^#^ *P* < 0.05 pre *vs*. post; ^§^ *P* < 0.05 group × time interaction.

### Total energy and macronutrient intake and appetite sensations

With the exception of the ketone ester/control drink and the post‐exercise recovery shakes prescribed by the study protocol, food intake during the full study period was *ad libitum*. Concurrent with the increase in training workload from training week 1 to 3, KE subjects spontaneously increased their total energy intake by ∼20% from ∼14 700 to ∼17 600 kJ/day. Total energy intake increased proportionately (+1966 ± 826 kJ/day at week 2 and +2880 ± 489 kJ/day at week 3, *P* < 0.01 and *P* < 0.001 *vs*. week 1, respectively) with the training load and the concomitant increase in energy expenditure [+1089 ± 135 kJ/day at week 2 and +3362 ± 104 kJ/day at week 3; assuming a mechanical efficiency of 23.8% (Ettema & Loras, 2009)] in KE, while it remained stable in CON (energy intake: −384 ± 794 kJ/day at week 2 and −157 ± 751 kJ/day at week 3, *P* = 0.86) (Table 4). The increasing energy intake in KE was largely effected by greater amounts of carbohydrate intake (+25.5 ± 6.3% at week 2 and +29.9 ± 5.9% at week 3, both *P* < 0.001) at fairly constant fat and protein intake. Subjective ratings of appetite were similar between the groups and over time (data not shown).

**Table 4:** Effect of ketone ester supplementation on total energy and macronutrient intake

	Control			Ketone ester		
	Week 1	Week 2	Week 3	Week 1	Week 2	Week 3
Energy intake	15,439 ± 741	15,055 ± 493	15,282 ± 808	14,688 ± 1042	16,655 ± 854#	17,568 ± 1121#
Carbohydrate	7211 ± 311	7412 ± 329	7417 ± 397§	6934 ± 354	8647 ± 464#	9014 ± 614# ^,^ §
Protein	2655 ± 228	2673 ± 65	2613 ± 163.	2807 ± 230	3019 ± 217	3273 ± 235
Fat	3362 ± 221	3293 ± 205	3394 ± 380	2878 ± 502	3240 ± 328	3413 ± 578

Data are mean ± SEM (kJ/day) for total energy and macronutrient intake, in subjects receiving either ketone ester supplements (*n* = 9) or control (*n* = 9). Macronutrient data are exclusive of the KE and CON drinks. ^#^ *P* < 0.05 *vs*. week 1; ^§^ *P* < 0.05 KE *vs*. CON at indicated time point.

### Hormonal parameters

**Figure 6:**
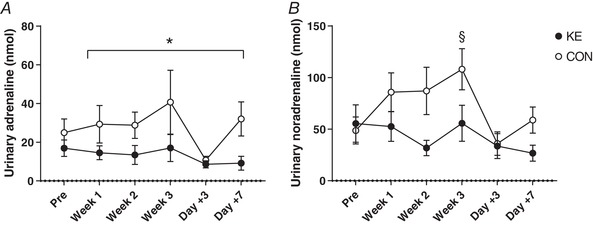
Effect of ketone ester supplementation on urinary catecholamine excretion Data are means ± SEM for urinary (*A*) adrenaline and (*B*) noradrenaline excretion before (Pre) and at the end of weeks 1 (Week 1), 2 (Week 2) and 3 (Week 3) of the training period, and after 3 (Day +3) and 7 (Day +7) days of recovery after training. Subjects received either control (○, *n* = 9) or ketone ester supplements (●, *n* = 9) following each training session. ^*^ *P* < 0.05 KE *vs*. CON; ^#^ *P* < 0.05 *vs*. PRE for both KE and CON; ^§^ *P* < 0.05 *vs*. PRE for indicated group.

**Figure 7:**
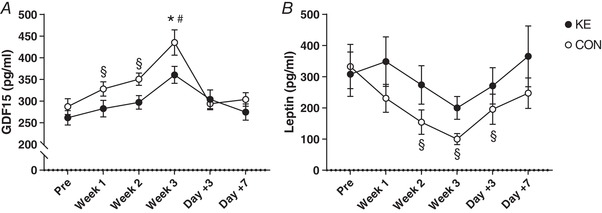
Effect of ketone ester supplementation on ‘appetite’ hormones involved in energy balance regulation Data are means ± SEM and represent changes in (*A*) GDF15 and (*B*) leptin concentration before (Pre), after 1 (Week 1), 2 (Week 2) and 3 (Week 3) weeks of training and following 3 (Day +3) and 7 (Day +7) days of recovery. Subjects received either control (○, *n* = 9) or ketone ester supplements (●, *n* = 9) during the training period. ^*^ *P* < 0.05 KE *vs*. CON; ^#^ *P* < 0.05 *vs*. PRE for both KE and CON; ^§^ *P* < 0.05 *vs*. PRE for indicated group.

We measured ‘energy homeostasis and appetite hormones’ (GDF15, leptin, ghrelin), urinary catecholamines and the ACTH–cortisol hypothalamic pituitary axis. All hormonal levels were similar between the groups at baseline (Figs 6 and 7). Serum GDF15 gradually increased during the training period in all subjects (*P* < 0.001), yet the rise was greater in CON than in KE (*P* < 0.05). Thus, in the post‐test serum GDF15 levels were markedly lower in KE (361 ± 19 pg/ml) than in CON (435 ± 29 pg/ml, 95% CI of KE *vs*. CON: −17 to −133 pg/ml, *P* < 0.05, *d* = 0.91). However, values rapidly returned to baseline during the recovery week. Compared to baseline, the 3‐week training intervention decreased serum leptin 3‐fold in CON (*P* < 0.001), but not in KE (*P* = 0.26), returning to baseline within 1 week after training. Nonetheless, serum leptin levels were not significantly different between the groups at any time. Serum ghrelin was not affected by the training programme or by ketone ester intake (KE_Pre_: 486 ± 38 pg/ml; KE_Post_: 471 ± 31 pg/ml; CON_Pre_: 466 ± 31 pg/ml; CON_Post_: 461 ± 30 pg/ml; group effect: *P* = 0.74; time‐effect: *P* = 0.32). Nocturnal urinary catecholamine excretions were stable throughout the full intervention period in KE. In contrast, in CON both the adrenaline and the noradrenaline excretions increased about 2‐fold from the pre‐test to the post‐test (main group effect for adrenaline, *P* < 0.01; interaction effect for noradrenaline, *P* < 0.01). Thus, in the post‐test urinary noradrenaline output was 108 ± 20 nmol in CON (*P* < 0.01 *vs*. pre‐test) and 56 ± 17 nmol in KE (*P* = 0.82 *vs*. pre‐test). Corresponding values for adrenaline excretion were 41 ± 17 nmol in CON *vs*. 17 ± 7 nmol in KE (group effect: *P* < 0.01). Plasma ACTH levels tended to decrease in both groups from the pre‐test to the post‐test (pre‐test 54.4 ± 6.4 ng/l *vs*. post‐test 44.1 ± 5.5 ng/l, *P* = 0.08), while serum cortisol levels were unchanged (pre‐test 184.5 ± 8.0 μg/l *vs*. post‐test 182.2 ± 7.8 μg/l, *P* = 0.83). ACTH/cortisol ratio tended to decrease in CON (pre‐test 340 ± 91 × 10^−6^ *vs*. post‐test 196 ± 22 × 10^−6^, *P* = 0.07), but not in KE (pre‐test 306 ± 46 × 10^−6^ *vs*. post‐test 278 ± 55 × 10^−6^, *P* = 0.72).

**Figure 8:**
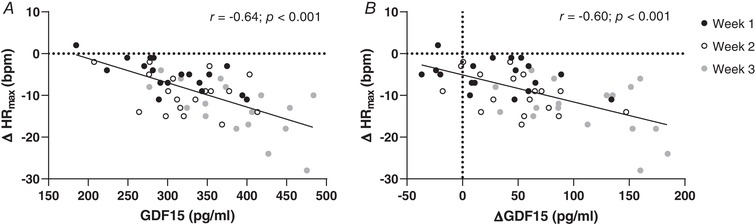
Relationship between GDF15 and maximal heart rate Correlation analyses showing a strong negative correlation between alterations in maximal heart rate (∆HR_max_) and both (*A*) absolute and (*B*) delta GDF15 during the training period. Individual data points represent changes after 1 (Week 1), 2 (Week 2) and and 3 (Week 3) weeks of training, compared to pre‐test values.

Moderate to strong negative correlations were found between both absolute and delta GDF15 levels and changes in maximal heart rate during the training period (*r* = −0.64 and *r* = −0.59, respectively, both *P* < 0.001). One outlier was identified in both data sets, but its removal only marginally affected the correlation coefficients (*r* = −0.64 and *r* = −0.60, respectively, both *P* < 0.001, Fig. 8). Changes in leptin levels were positively correlated with alterations in BMC (*r* = 0.56, *P* < 0.05).

### Cytokine and immune response

Plasma IL‐6 levels were similar between both groups in the pre‐test (KE: 0.68 ± 0.06 *vs*. CON: 0.85 ± 0.12 pg/ml; *P* = 0.71) and were stable throughout the study in both groups (data not shown). Baseline values for lymphocyte subtype counts, i.e. CD3^+^, CD4^+^ and CD8^+^, were similar between the groups (Table 5). Compared to the pre‐test, in the post‐test CD3^+^ and CD8^+^ cell counts were decreased by ∼5 and 14%, respectively (*P* < 0.001), whereas CD4^+^ increased by ∼6% (*P* < 0.001). As a result CD4^+^/CD8^+^ ratio increased by ∼25% (*P* < 0.001). Lymphocyte changes were similar between the groups.

**Table 5:** Effect of ketone ester supplementation on lymphocyte cell counts

	Control		Ketone ester	
	Pre	Post	Pre	Post
Mature T (CD3^+^)	1.13 ± 0.02	1.07 ± 0.01#	1.11 ± 0.02	1.06 ± 0.01#
T‐helper (CD4^+^)	0.57 ± 0.02	0.59 ± 0.02#	0.56 ± 0.04	0.60 ± 0.03#
T‐cytotoxic (CD8^+^)	0.48 ± 0.03	0.41 ± 0.02#	0.43 ± 0.03	0.37 ± 0.02#
CD4^+^/CD8^+^	1.23 ± 0.09	1.49 ± 0.12#	1.35 ± 0.14	1.67 ± 0.15#

Data are means ± SEM (*n* × 10^9^ cells/l) for lymphocyte subtype counts in subjects receiving either ketone ester supplements (*n* = 9) or control (*n* = 9). ^#^ *P* < 0.05 pre *vs*. post.

### Bone metabolism markers

The training period decreased total serum osteocalcin concentration by ∼30% (pre‐test: 19.5 ± 0.6 ng/ml *vs*. post‐test: 13.1 ± 0.6 ng/ml, *P* < 0.05), irrespective of the experimental condition. Levels returned to baseline within the 1‐week recovery period. TRAP5b activity was not modified by the training or by ketone ester intake (KE_Pre_: 4.25 ± 0.25 U/l; KE_Post_: 4.23 ± 0.23 U/l; CON_Pre_: 4.06 ± 0.36 U/l; CON_Post_: 3.92 ± 0.26 U/l; group effect: *P* = 0.30; time effect: *P* = 0.18). Alterations in osteocalcin showed a strong inverse correlation with changes in GDF15 (*r* = −0.61, *P* < 0.01).

### Blood glucose levels and muscle glycogen content

Initial muscle glycogen content was 140 ± 8 mmol/kg wet weight in CON *vs*. 122 ± 9 mmol/kg in KE (*P* = 0.41). The training period slightly decreased resting muscle glycogen content in CON (−28 ± 9 mmol/kg, *P* < 0.05) but not in KE (+2.4 ± 13.2 mmol/kg, *P* = 0.99), yielding identical muscle glycogen contents between CON (111 ± 10 mmol/kg) and KE (124 ± 9 mmol/kg) in the post‐test. The TT_30min_ decreased muscle glycogen content in both groups by ∼44% during the pre‐test (KE: 59 ± 10 mmol/kg; CON: 69 ± 12 mmol/kg; both *P* < 0.001 compared to pre‐exercise), while muscle glycogen was unaltered by the TT_30min_ in the post‐test (KE: 92 ± 9 mmol/kg; CON: 86 ± 8 mmol/kg; *P* = 0.10 and *P* = 0.19 *vs*. pre‐exercise, respectively) for both groups. Plasma glucose levels immediately before the TT_30min_ were similar between the groups during both the pre‐ and the post‐test (KE_Pre_: 4.1 ± 0.3 mm; KE_Post_: 4.9 ± 0.2 mm; CON_Pre_: 3.9 ± 0.2 mm; CON_Post_: 4.2 ± 0.4 mm; group effect: *P* = 0.45; time effect: *P* = 0.37). At the end of the TT_30min_, glucose levels were increased by ∼1.5 mm (*P* < 0.001 *vs*. pre‐exercise) during both the pre‐ and the post‐test independent of KE supplementation (KE_Pre_: 5.6 ± 0.3 mm; KE_Post_: 5.9 ± 0.2 mm; CON_Pre_: 6.0 ± 0.4 mm; CON_Post_: 5.3 ± 0.6 mm).

### Stress–recovery state

The training period substantially decreased the stress–recovery state in both groups (pre‐test: 80 ± 5 *vs*. post‐test: −6 ± 7, *P* < 0.001). This was due to an increase in ‘total stress’ scores (pre‐test: 49 ± 3 *vs*. post‐test: 110 ± 6, *P* < 0.001) together with a decrease in ‘total recovery’ scores (pre‐test: 129 ± 4 *vs*. post‐test: 104 ± 4, *P* < 0.001). Stress‐recovery state rapidly improved during the recovery week, yet values remained below baseline even at day 7 (69 ± 6, *P* < 0.05 *vs*. pre‐test). There were no differences between the groups at any time.

### Gastrointestinal tolerance

Gastrointestinal discomfort scores were slightly higher in the post‐test than in the pre‐test (pre‐test: 7 ± 1 *vs*. post‐test: 11 ± 2 out of a maximum of 96, *P* < 0.05). This small increase was due to a higher incidence of lower abdominal symptoms (pre‐test: 2 ± 1 *vs*. post‐test: 4 ± 1 out of a maximum of 32, *P* < 0.05) and more systemic discomfort (pre‐test: 3 ± 1 *vs*. post‐test: 4 ± 1 out of a maximum of 32, *P* < 0.05). Upper abdominal distress was stable between the pre‐test and post‐test (*P* = 0.73). Gastrointestinal discomfort scores returned to baseline within the 7‐day recovery period. No differences were observed between the experimental conditions at any time point.

### Identification of experimental condition

Upon completion of the study the subjects were asked to identify their presumed group allocation. Nobody was certain of his experimental condition, but 10 out of 18 (5 in KE and 5 in CON) subjects correctly guessed the condition based on subjective perceptions, indicating successful blinding of the treatments.

## Discussion

Early detection and prevention of overtraining is pivotal in athlete training management. From this perspective we investigated whether post‐exercise ketone ester intake (KE) can prevent non‐functional overreaching and performance impairment during an episode of excessive training load. The endurance training programme used induced explicit cardiovascular, hormonal and perceptual symptoms of overreaching in all subjects. Interestingly, KE markedly inhibited the appearance of these symptoms, whilst enhancing tolerable training load, increasing energy intake and stimulating endurance exercise performance. Our data indicate that KE is a potent strategy to prevent overtraining and stimulate endurance training adaptation. In addition, we provide preliminary evidence that GDF15 may be a valid hormonal marker of overtraining.

Although the complex pathophysiology is still poorly understood, it is the prevailing opinion that autonomic neural imbalance plays an important role in the development of overtraining (Fry *et al*. 1991; Lehmann *et al*. 1998). From this perspective two distinct overtraining types were defined. ‘Sympathetic’ overtraining is characterized by elevated basal sympathetic tone, whilst sympathetic drive is abnormally decreased in the ‘parasympathetic’ overtraining form (Israel, 1976). The former has primarily been associated with high‐intensity anaerobic exercise, *versus* the latter with endurance training activities (Kuipers & Keizer, 1988), but both forms probably rather exist as a continuum (Fry *et al*. 1994a, 2006). Initially sympathetic overtraining develops as a stress response attempting to maintain functional status. However, in a later stage, parasympathetic symptoms gradually predominate due to fatigue at the site of the sympathetic neuroendocrine system. In the CON conditions of the current study, the subjects exhibited the initial basal sympathetic stress response as evidenced by elevated nocturnal catecholamine excretion, predominantly noradrenaline, which reflects spillover from the sympathetic nervous system (Esler *et al*. 1988; Fry *et al*. 1994a). Nonetheless, against the face of this elevated sympathetic activity, resting heart rate decreased significantly, which indicates that at the cardiac site the elevated central sympathetic drive was probably overruled by excess vagal output (Hedelin *et al*. 2000; Pichot *et al*. 2002) either or not in conjunction with downregulation of cardiac β_2_‐adrenoceptor sensitivity (Gleeson, 2002; Fry *et al*. 2006). However, these autonomic responses were substantially altered by KE during training. KE fully counteracted the initial training‐induced sympathetic overdrive, as evidenced by stable nocturnal catecholamine excretion throughout the full training period. Nonetheless resting heart rate slightly increased in weeks 1 and 2, probably indicating transient sympathetic dominance at the site of the heart. KE intake also markedly impacted exercise tachycardia, which also depends on sympathetic–parasympathetic interactions. In CON the training markedly suppressed the exercise‐induced rise in heart rate, including a substantial drop in maximal heart rate by 10–28 bpm. KE administration clearly counteracted this effect, probably due to sympathetic regulation (Lehmann *et al*. 1991; Stanojevic *et al*. 2013). However, we did not measure plasma catecholamine levels during exercise. Nevertheless, central autonomic command of exercise heart rate is also controlled by input from mechano‐ and metaboreceptors in active muscles (Fisher, 2014), which might also be changed by KE intake. Furthermore, regulation might also occur at the site of cardiac adrenergic sensitivity or G protein‐coupled receptor 41 (GPR41) activity. GPR41 is present in sympathetic ganglions and is thereby directly involved in sympathetic control (Kimura *et al*. 2011). d‐βHB suppresses GPR41 activity in mice, resulting in a depressed sympathetic tone and heart rate (Kimura *et al*. 2011).

Apart from the KE or CON supplements, the subjects received a standardized protein–carbohydrate solution to stimulate recovery immediately after each training session. Intake of other foods and drinks was *ad libitum* because we wanted to evaluate the effect of KE on appetite regulation and spontaneous food intake. It is well known that overtraining in endurance athletes often results in appetite suppression (Fry *et al*. 1992). Furthermore, decreased appetite during the ketogenic diet has been linked to elevated plasma ketone levels (Paoli *et al*. 2015). However, in the current study, neither the training overload nor KE decreased appetite/hunger perception. Stubbs *et al*. (2018) have demonstrated that acute KE intake, which elevated plasma d‐βHB levels to ∼3–4 mm compared to isocaloric glucose ingestion, suppressed appetite in healthy subjects. However, appetite scoring in the current study was done in the early morning in the fasted state when blood d‐βHB levels were basal in both experimental groups (<0.4 mm). Nonetheless, actual food intake pattern was significantly different between CON and KE. Spontaneous energy intake in CON was constant throughout the study (∼15 000 kJ per day) despite the gradual increase in training load from week 1 to week 3 (see Fig. 3), resulting in an energetic deficiency of ∼1470 and ∼2800 kJ per day during weeks 2 and 3, respectively. Conversely, KE gradually increased energy intake to ∼17 600 kJ per day, predominantly via extra carbohydrate intake, resulting in an energetic balance during both week 2 and week 3 (average energy surplus of 198 kJ per day). It is important to note that the effect of KE to blunt the appearance of overreaching symptoms such as decreased heart rates, elevated urinary noradrenaline excretion and elevated serum GDF15 level was obvious before any significant difference in daily energy intake occurred. This suggests that modulation of food intake per se was not the primary mechanism of action of KE. Aiming to elucidate the underlying mechanism, we measured serum levels of the ‘appetite hormones’ leptin, ghrelin and GDF15. GDF15 is a peptide which has only recently been discovered to act as a stress‐induced hormone that is involved in appetite regulation by decreasing food intake (Johnen *et al*. 2007; Macia *et al*. 2012; Wang‐Wei Tsai *et al*. 2013; Patel *et al*. 2019). Here, for the first time, we demonstrate that training overload increased systemic GDF15 level. Interestingly, this effect was negated by KE intake, which might at least partly explain the higher energy intake during training in the latter group. This observation together with literature data (Patel *et al*. 2019) indicates that the GDF15 increment in CON reflects training‐induced physiological stress, rather than a moderate energy deficit. Additional support for this comes from a recent study in our laboratory showing that 4 weeks of hypocaloric diet (30% energy deficit), aimed to induce body weight loss (minus 2–4 kg) in fit lean females, did not alter systemic GDF15 levels either in the presence or in the absence of KE supplementation (Hiroux *et al.*, unpublished observations). Note that this suppression of GDF15 occurred in the absence of elevated plasma d‐βHB concentration, indicating adaptation of GDF15 secretion by short‐term KE intake. Conversely, neither leptin nor ghrelin were directly involved in food intake regulation during the intervention period. Serum leptin, an appetite suppressor, was even higher during training in KE than in CON, while ghrelin was unaffected. However, it is well established that leptin during episodes of energy deficit operates as a ‘starvation signal’ by decreasing basal energy expenditure through suppression of heart rate, blood pressure, thyroid hormone levels and sympathetic nervous system activity (Flier, 1998; Pandit *et al*. 2017). Thus, lower basal metabolic rate effected by a decrease in leptin (Woods et al. 2017, 2018) might explain the absence of body weight drop in CON despite energy expenditure in training exceeding energy intake during the later stage of the training period.

It is also well known that the dysregulation of hormonal and energy balance in overtraining, especially in non‐weight‐bearing sports such as cycling and swimming, can stimulate bone demineralization and thereby impair long‐term bone health (Nagle & Brooks, 2011; Olmedillas *et al*. 2012). In both experimental groups, we observed a decrease of the bone formation marker osteocalcin against stable activity of the osteoclast marker TRAP5b. This may indicate net bone resorption (Crockett *et al*. 2011; Ferreira *et al*. 2015). These results are in accordance with a study showing similar symptoms of net bone resorption occurring in professional cyclists during a 3‐week cycling race, i.e. Giro d'Italia (Lombardi *et al*. 2012). Nonetheless, the training intervention did not significantly alter BMD or BMC measured by a whole‐body DXA scan. This may be due to the short duration of the intervention, because months rather than weeks of strenuous cycling training are needed to induce a measurable degree of bone demineralization (Barry & Kohrt, 2008). In addition, consistent ingestion of a protein–carbohydrate mixture may be sufficient to maintain bone mineral status (Townsend *et al*. 2017). Other studies also have suggested a link between bone metabolism and energy balance regulation (Confavreux *et al*. 2009; Lombardi *et al*. 2012). Along such interaction, ∆leptin during training was positively correlated with ∆BMC (*r* = 0.56). In addition, a training‐induced rise of GDF15 was associated with a decrease in osteocalcin (*r* = −0.61), which for the first time indicates that GDF15 may be implicated in the regulation of bone metabolic activity. Taken together, the above observations support a tight link between maintenance of energy balance and bone metabolism during intensified training. In fact, the beneficial effect of KE on bone mineral content during the overtraining period might be largely explained by better matching of energy intake to energy expenditure in training driven by the concerted actions of appetite hormones, most prominently GDF15.

It is well established that low muscle glycogen levels in endurance athletes impair the capacity to sustain strenuous training. Based on longitudinal observations in endurance (over)trained individuals it has been postulated that glycogen depletion may be involved in the development of overtraining (Costill *et al*. 1971, 1988). Against such an opinion, our current observations show that despite a multiplicity of other symptoms of overreaching, resting glycogen levels remained within the normal range in both groups, confirming previous studies showing that glycogen depletion is not a prerequisite for overtraining to develop (Snyder *et al*. 1995; Halson *et al*. 2004). In addition, pre‐ and post‐exercise muscle glycogen contents were similar between CON and KE. Furthermore, in line with earlier findings (Achten *et al*. 2004), the training overload markedly blunted exercise‐induced net muscle glycogen breakdown, which has been attributed to decreased adrenergic sensitivity (Lehmann *et al*. 1993; Jeukendrup & Hesselink, 1994). Again, KE did not impact exercise‐induced net glycogen breakdown during a 30 min maximal exercise bout. However, the training intervention also substantially reduced peak blood lactate levels produced by a 90 s all‐out exercise bout. This effect faded within 3 days of recovery in CON, whilst persisting in KE until day +7.

The overload training programme elicited clear physiological dysregulations as well as negated positive training adaptations, which clearly indicates a state of functional overreaching in some, *versus* non‐functional overreaching in others. Sprint performance decreased in all subjects (range: −1 to −12%), whilst endurance performance in TT_30min_ on average was stable (range: −4 to +12%), which corroborates earlier findings (Woods *et al*. 2018). Sprint performance restored to baseline within 3 days of recovery, while performance improvements in TT_30min_ only occurred by day +7. Nonetheless, in some subjects performance impairments persisted until the end of the recovery period (90S: −5 to +13%; TT_30min_: −5 to +14%). KE administration did not alter sprint power output, but clearly stimulated endurance exercise performance during the final week of the overload period, as evidenced by a 15% increase in both training load and EPT_120min_ compared to CON. Note that the observed increase in endurance performance during the final stage of the training period coincided with higher rate of daily carbohydrate intake in KE than in CON. However, this caused neither different blood glucose levels nor different muscle glycogen contents between the groups. Nonetheless, it cannot be excluded that beneficial regulation of blood glucose turnover during exercise contributed to explaining the ergogenic effect of KE in EPT_120min_.

The training overload programme induced many responses indicating the development of a physiological overtraining status. Concomitantly, mental well‐being consistently deteriorated. Results from the RESTQ‐76 Sport questionnaire indicate impaired general well‐being, attention issues, and both physical and emotionally exhaustion. The decrement in RESTQ‐76 scores in fact was similar to the effect of a 4‐week overload training period in well‐trained triathletes (Coutts *et al*. 2007), but was independent of the experimental group. This shows that in the conditions of the current study RESTQ‐76 Sport scoring was less sensitive than the physiological measures in identifying the development of overtraining.

The primary aim of the study was to assess the effect of KE in overtraining. At the same time, this is the first longitudinal study to explore such a wide spectrum of physiological responses induced by a period of deliberate and well‐controlled endurance training overload. Our observations corroborate the prevailing opinion that overtraining is a complex integrative response involving dysregulations in multiple systems, including the autonomous nervous system, energy balance and hormonal status (Lehmann *et al*. 1998; Kreher & Schwartz, 2012), which eventually also result in immunological impairment. Therefore, identification of a harmful degree of overreaching by a single biomarker is conceivably irrelevant (Urhausen & Kindermann, 2002; Hecksteden *et al*. 2016; Greenham *et al*. 2018). Attempts to use hormonal markers of overtraining, such as ACTH, cortisol, growth hormone, thyroid hormones and prolactin, have generally failed. Nonetheless, here we show GDF15 to consistently increase with training overload. Furthermore, GDF15 changes clearly discriminated the less (KE) *versus* the more overtrained (CON) experimental group, and both delta and absolute GDF15 was highly correlated with the training‐induced drop of peak exercise heart rate (*r* = 0.60 and *r* = 0.64, respectively). Moreover, our data indicate that GDF15 is not only implicated in the regulation of energy balance during training, but is probably also implicated in the regulation of bone metabolism. Clearly GDF15 deserves further attention as a potential sensitive hormonal marker of overtraining development.

Previous studies have raised concerns about the implementation of ketone supplementation because of a high incidence of gastrointestinal symptoms due to acute ketone ingestion (Leckey *et al*. 2017; Vandoorne *et al*. 2017). However, compared to ketone ester intake, gastrointestinal symptoms are more explicit with ingestion of ketone salts (Veech, 2004). In the conditions of the current study, involving KE three times daily, the incidence of gastrointestinal complaints was low and similar between KE and CON. This suggests that gastrointestinal distress was due to the overtraining per se (De Oliveira & Burini, 2009) rather than to the supplements.

In conclusion, here we demonstrate that an oral ketone ester is a potent nutritional strategy that prevents the development of physiological overtraining symptoms and non‐functional overreaching. In addition, we provide preliminary observations to indicate that GDF15 may be an adequate hormonal marker for the development of overreaching/overtraining.

## Additional information

### Competing interests

The authors declare that they have no competing interests.

### Author contributions

Conception and design of the study: CP and PH. Biopsy sampling: RVT. Data collection and biochemical analyses: CP and MR. Analysis and interpretation of the data and manuscript drafting: CP and PH. All authors critically evaluated the manuscript and approved it for submission. All authors agree to be accountable for all aspects of the work in ensuring that questions related to the accuracy or integrity of any part of the work are appropriately investigated and resolved. All persons designated as authors qualify for authorship, and all those who qualify for authorship are listed.

### Funding

This study was funded by Research Fund Flanders (Fonds voor Wetenschappelijk Onderzoek – Vlaanderen; research grant no. G080117N).
